# Digital games and virtual reality applications in child abuse: A scoping review and conceptual framework

**DOI:** 10.1371/journal.pone.0276985

**Published:** 2022-11-09

**Authors:** Afsoon Asadzadeh, Hassan Shahrokhi, Behzad Shalchi, Zhila Khamnian, Peyman Rezaei-Hachesu

**Affiliations:** 1 Student Research Committee, Tabriz University of Medical Sciences, Tabriz, Iran; 2 Department of Health Information Technology, School of Management and Medical Informatics, Tabriz University of Medical Sciences, Tabriz, Iran; 3 Research Center of Psychiatry and Behavioral Science, Tabriz University of Medical Sciences, Tabriz, Iran; 4 Department of Psychiatry, Tabriz University of Medical Sciences, Tabriz, Iran; 5 Department of Community Medicine, Tabriz University of Medical Sciences, Tabriz, Iran; Sreenidhi Institute of Science and Technology, INDIA

## Abstract

Child abuse refers to any form of maltreatment, including physical abuse, sexual abuse, emotional abuse, and neglect that occurs to children under 18 years of age. Digital games and virtual reality (VR) can be used as beneficial solutions for dealing with child maltreatment concerns. This study aimed to present a conceptual framework for showing the applications of these technologies in managing child abuse. The framework is developed in two stages: (1) a scoping review to gather digital games and VR applications for child abuse issues through the search in PubMed, Ovid (APA PsycInfo), Scopus, Web of Science, ProQuest, Institute of Electrical and Electronics Engineers (IEEE), Cochrane Database of Systematic Reviews, and grey literature and (2) developing a conceptual framework based on the review results and validating it by 12 experts. The proposed conceptual framework shows that digital games and VR have been used for six main topics: (1) medical education, (2) prevention, (3) screening, (4) diagnosis, (5) treatment, and (6) forensic medicine in response to child abuse issues. Studies have more focused on child sexual abuse prevention, behavioral monitoring of sexual offenders in forensic medicine, and knowledge or performance assessment of students in medical education. Serious games (SGs), computer simulation, and immersive VR were common technologies for children, students, and forensic medicine, respectively. The experts believe the combination of immersive features of VR with SGs can further encourage user engagement. It appears that digital games and VR can play a positive role in child abuse management. Given the extensive capabilities of these technologies, further studies are needed to show all their potential applications for child abuse problems.

## Introduction

Child abuse as a major public health concern refers to any form of maltreatment by an adult that occurs to children under 18 years of age [1, 2]. It may cause actual or potential harm to the child’s health, survival, development, or dignity in the context of a responsible, trusting, or power relationship. Four common types of child maltreatment are recognized in the literature, i.e., 1) emotional or psychological abuse, 2) child neglect, 3) physical abuse, and 4) sexual abuse [3, 4]. Knowledge of risk factors and their consequences is critical to managing and preventing violence against children [5].

There are various risk factors for child abuse that can be classified into four groups, thus; 1) individual level (e.g., child age, child sex, children with special healthcare needs or disabilities), 2) interpersonal level (e.g., well-established family-level risk factors such as poverty, substance use disorders, and mental health disorders), 3) community level (e.g., neighborhood crime and violence), and 4) societal level (e.g., specific economic policies and trends, and taxes that take a larger proportion of income) [6]. Child abuse may have various short-term and long-term health consequences [7, 8]. For example, it can lead to inter-personal problems, high anxiety, depression, Post-Traumatic Stress Disorder (PTSD), low self-esteem, substance abuse, suicide, obesity, high-risk behavior, inconsistent attendance and performance at school, signs of general neglect, and attention-seeking behavior [4, 7, 9].

Appropriate solutions for mitigating and managing child maltreatment are required [10]. Accordingly, technology-based innovations may play a beneficial role in this social concern [11]. In other words, the use of some information technology (IT) applications can be useful in reducing child vulnerabilities and other major consequences in emergency settings [12]. For example, SafeCare technology has been developed for parents to prevent child maltreatment and neglect by improving behavioral education using tablet-based software [11]. Additionally, Virtual reality (VR) and digital games (e.g., serious games (SGs) and gamification) as innovative digital technologies have the potential to respond to child abuse concerns [13–15].

VR is a new form of the human-computer interface that can provide a virtual, interactive, and immersive world using special electronic equipment and software [16]. SG is defined as any game that is used for purposes other than entertainment, such as education, prevention, and treatment, as well as, gamification was developed to “the use of game design elements within non-game contexts” [17, 18]. Studies have shown applications of these technologies in the management of child abuse [15, 19–21]. For instance, Malamsha et al. (2021) have developed a mobile-based SG to prevent child sexual abuse (CSA) based on the sociocultural context of Tanzania [20]. Pan et al. (2018) designed the immersive VR system to test whether the level of professional experience and cognitive load have any impact on a GP’s ability to correctly identify (and devise some strategy to address) child safeguarding concerns [21].

Therefore, concerns about child abuse have led to the development of IT-based solutions to help in preventing or reducing child maltreatment consequences. Given the emerging IT solutions, digital games and VR have been used in various aspects of child abuse issues, such as prevention and education. Currently, there is no research focusing on the applicability of these technologies for child maltreatment. Providing the conceptual framework for managing child abuse based on mentioned technologies will help inform policymakers, government, psychiatrists, psychologists, informatics, health information management, and health information technology experts on how to use and plan the digital game and VR-based solutions for the child maltreatment problems. Consequently, this study aimed to present a conceptual framework to determine the applications of digital games and VR in the management of child abuse.

## Materials and methods

### Ethics statement

The study was approved by the Tabriz University of Medical Sciences Ethics Committee based on a proposal leading to a Ph.D. thesis (Number: IR.TBZMED.REC.1401.465). Additionally, it is a part of the CHATR (Child Abuse & Trauma Research) project. CHATR is a comprehensive research of the Tabriz University of Medical Sciences for managing various aspects of child abuse at the individual, family, and community levels (Number: IR.TBZMED.REC.1395.1182). Invited participants (i.e., psychiatry, psychology, health information technology/health information management, informatics, and community medicine experts) provided written informed consent through Google form to participate in this study. The consent form included information about the purpose of this study, potential benefits, and a respondent’s right to take part in the research or to withdraw from it at any time, without reprisal. This consenting procedure was approved by the Tabriz University of Medical Sciences Ethics Committee.

### An overview of the methods’ steps

The methodology of this study comprises two stages. First, a scoping review was performed to show VR or digital games (i.e., computer games, SGs, and gamification) solutions used for child abuse. The review was not registered and a protocol was not prepared. Second, the authors proposed an initial framework based on the findings of the previous phase, which was then sent to related experts (i.e., psychiatry, psychology, health information technology/health information management, informatics, and community medicine experts) for validation and completion.

### Phase 1. Scoping review

#### Databases and search strategy

The review was carried out in accordance with PRISMA for the scoping review. The PRISMA-ScR checklist is available as **S1 Table**. The keywords were extracted from the search strategy of various review papers which were conducted for child abuse, VR, and SG objectives. Additionally, psychiatry, psychology, health information technology/management, and medical informatics experts’ advice (n = 4) were used to select the keywords. An expert librarian confirmed the search strategy. Then, it was searched in the following electronic sources on November 23, 2021: PubMed (Medline and PMC), Ovid (APA PsycInfo), Scopus, Web of Science, ProQuest (Dissertation & Thesis Global Databases), Institute of Electrical and Electronics Engineers (IEEE), Cochrane Database of Systematic Reviews, and grey literature (i.e., opengrey.eu, Google Scholar, and manual search). The search details in PubMed have been shown in **S2 Table**.

#### Eligibility criteria

Studies were included if they met the following criteria: a) the intervention was VR or digital games (i.e., serious game, gamification, video game, computer game); b) studies that aimed at child abuse issues, c) peer-reviewed studies, and d) English papers. Studies were excluded according to the following criteria: a) child age > 18, b) protocols, c) review papers, d) letters to the editor, e) unrelated studies to the topic, and e) lack of access to full-text articles.

#### Study selection and data extraction

The references identified from the databases were imported into an EndNote X8 library, and the duplicates were removed. The papers were searched according to the inclusion criteria in three steps, including 1) title screening, 2) abstract screening, and 3) full-text screening. Disagreements items were resolved by consensus or through consultation with the third author. After selecting the related papers, the following data was extracted: authors (year), country, child abuse type, product type, study design, mean age of samples (M) and Standard Deviation (SD), assessment variables, intervention duration, main results, and technical aspects of the included papers (see **S3 and S4 Tables**).

### Phase 2. Proposed initial conceptual framework and validation

The results of Phase 1 were classified into sub-themes based on the digital games and VR applications for child maltreatment. Then, it was sent to experts with relevant experience, including psychiatrists (N = 3), psychologists (N = 3), social medicine specialists (N = 3), medical informatics (N = 2), and health information technology/management experts (*N* = 5), who were asked to confirm and apply their recommendations within the proposed conceptual framework for completion. The framework was revised and completed in response to expert comments, and the framework’s vulnerabilities were addressed.

## Results

### Phase 1. Scope of digital games and virtual reality for child abuse

**Fig 1:**
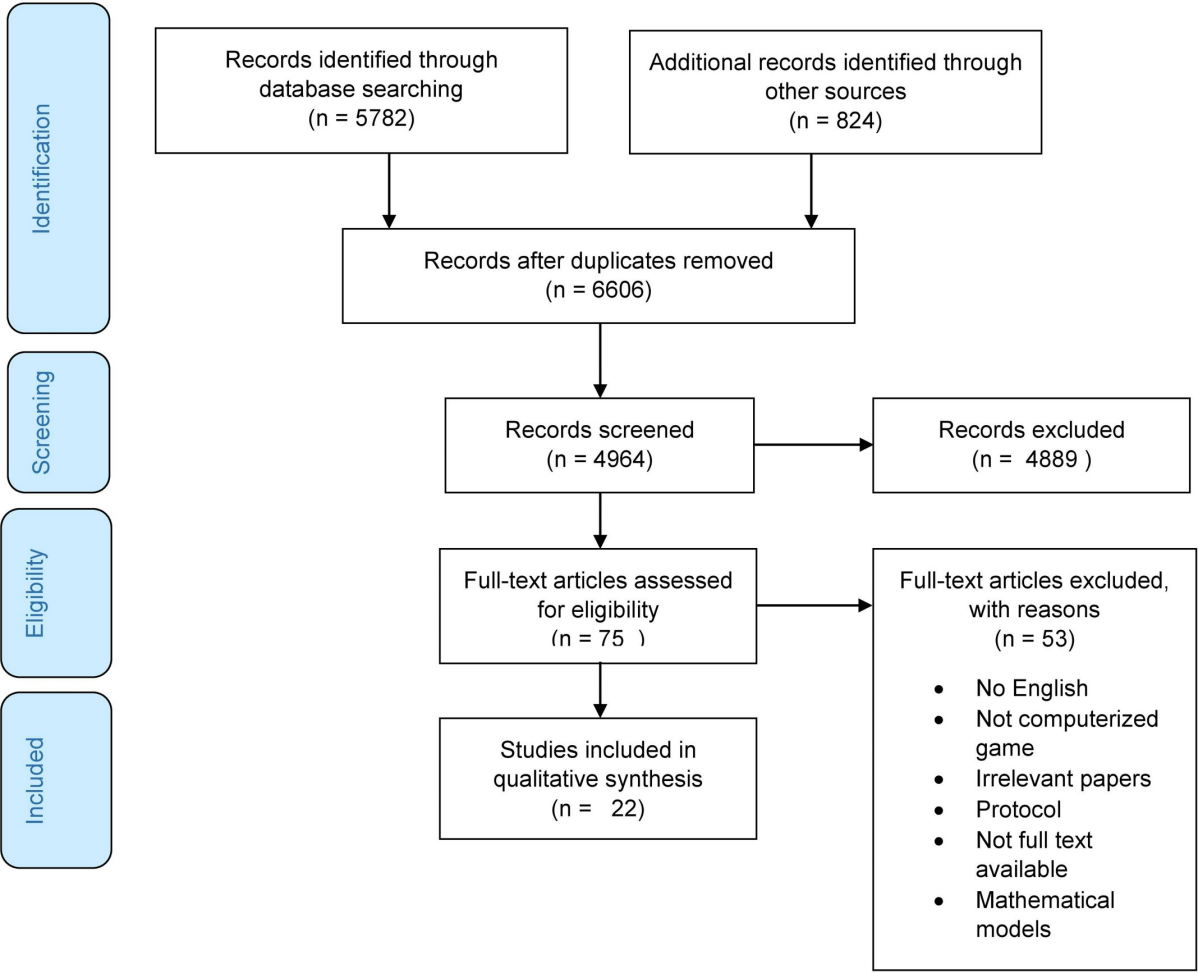
Flowchart for the selection process of the included studies.

**Fig 2:**
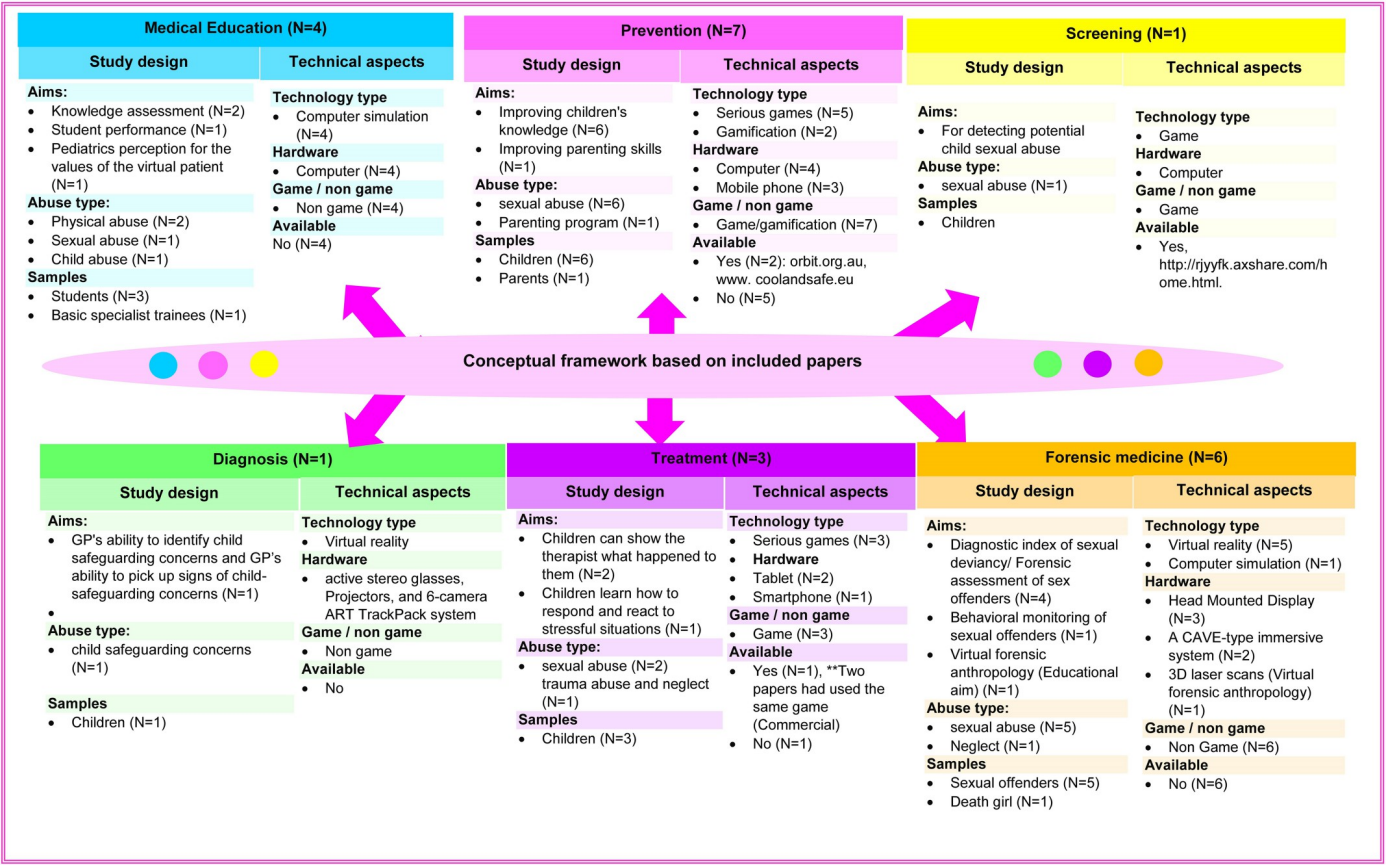
Overview of the digital games and VR for child abuse issues according to literature; N = Number of studies.

A total number of 6606 records were identified from different databases and gray literature. After removing duplicates, 4964 records remained. A total number of 75 papers were assessed for eligibility, of which 22 records meet the inclusion criteria (See Fig 1 for details). S3 and S4 Tables present the fundamental characteristics and technical aspects of the included studies reporting digital games and VR for child abuse issues, respectively. Finally, the applications of these technologies for child abuse issues were summarized in a taxonomy (see Fig 2).

According to included papers (see **S3 and S4 Tables**), the applications of VR and digital games for the child abuse are summarized into six core topics, i.e., 1) medical education, 2) prevention, 3) screening, 4) diagnosis, 5) treatment, and 6) forensic medicine. These topics and their subgroups are shown in the following taxonomy.

### Phase 2. The proposed conceptual framework for VR and digital games applications for child abuse

**Fig 3:**
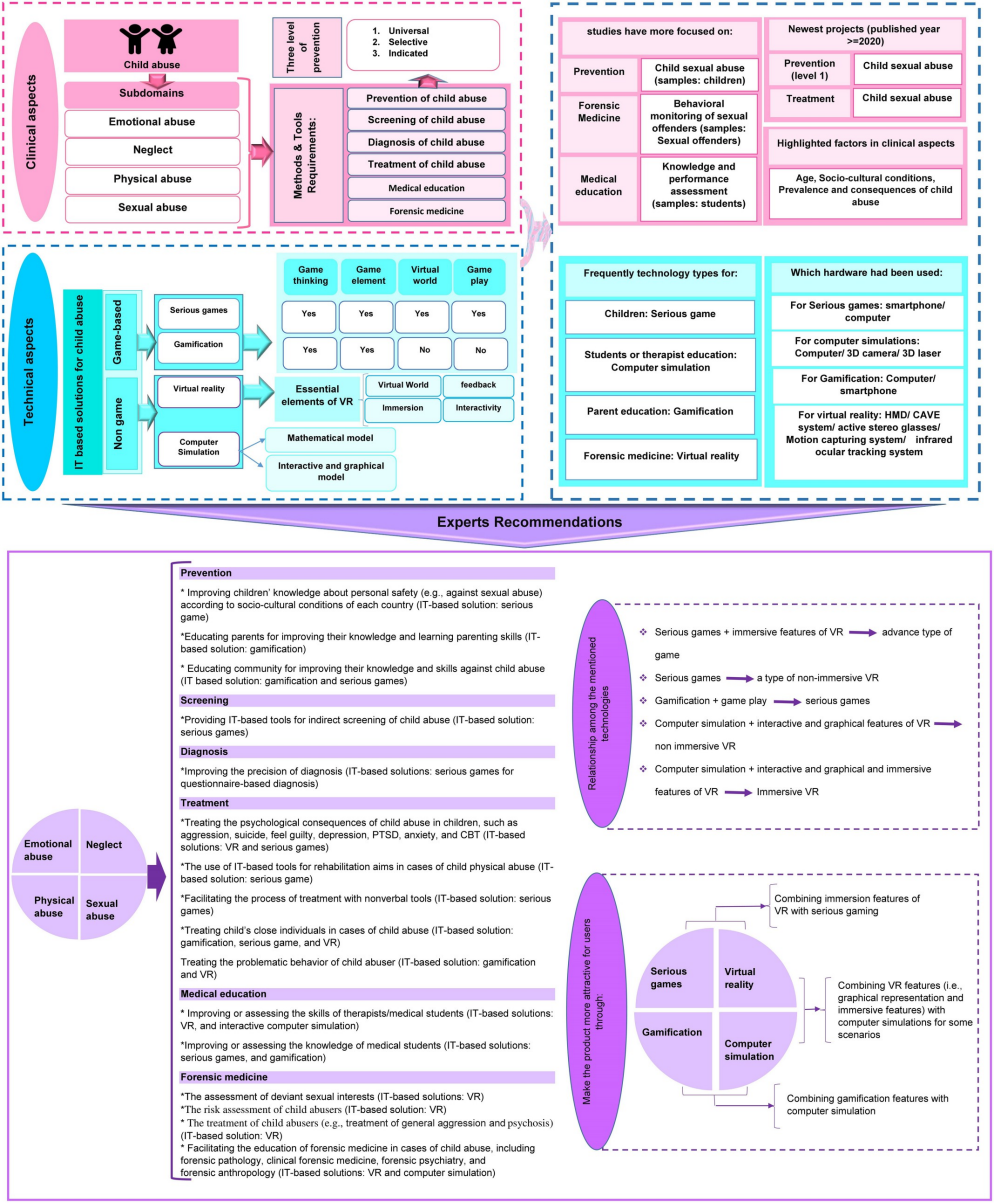
Conceptual framework for digital games and VR potentials in child abuse; IT: Information technology, VR: Virtual reality, PTSD: Post-traumatic stress disorder, CBT: Cognitive behavioral therapy.

As shown in Fig 3, a conceptual framework for the applications of VR and digital games in child abuse issues was proposed based on the results of the review and experts’ knowledge. The main components of the findings were presented into three main topics, including 1) clinical aspects, 2) technical aspects, and 3) expert recommendations. The clinical aspects show that all four types of child abuse (i.e., physical abuse, sexual abuse, emotional abuse, and neglect) need tools and methods for prevention, screening, diagnosis, treatment, medical education, and forensic medicine. In the technical aspects, IT-based solutions for managing child maltreatment using VR and digital games have been demonstrated. Then, for the applications of these technologies in cases of child abuse, evidence-based topics have been presented for clinical and technical aspects (e.g., which clinical or technical aspects have been more highlighted in the literature). Concerning the expert recommendations section, only 12 out of 16 experts (i.e., psychiatry (N = 3), health information management/health information technology (N = 5), informatics (N = 2), psychology (N = 1), and social medicine (N = 1) participated in this study to complete and validate the framework. Clinical experts recommended “which child abuse issues?” can be addressed using VR or digital games, and technical experts indicated “which types of IT-based tools?” can be used as appropriate solutions for addressing the child maltreatment issues in terms of six core topics. Expert recommendations were determined based on the end-users (e.g., children, parents, students, and therapists), the child abuse subdomains/requirements, and the accessibility of mentioned technologies. For instance, experts believe that SGs can be a suitable solution for children to help and prevent child sexual abuse. Additionally, they indicated some recommendations to create more attractive products for users (See Fig 3 for details).

## Discussion

The present study conducted a comprehensive review to show the applications of VR and digital games for managing child abuse issues. Then, a conceptual framework is proposed based on a comprehensive review and experts’ knowledge. Digital games and VR applications used against the child abuse are categorized into six main topics in our proposed conceptual framework, namely: 1) medical education, 2) prevention, 3) screening, 4) diagnosis, 5) treatment, and 6) forensic medicine [15, 19–40].

For medical/health education, the interactive and graphical simulation was used to assess students’ knowledge and performance in child maltreatment scenarios [19, 22–24]. According to a review study, computer-based simulations have positive effects on skill and/or knowledge acquisition in the educational process [41]. Ward et al. (2019) showed that summative evaluation in healthcare using computer simulations in the form of clinical scenarios was used to evaluate higher-order competency characteristics [42]. It should be noted that SGs or gamification can be used as an effective technology to improve knowledge, skills, and satisfaction in the medical education field [43]. Yasin and Abbas (2021) in their systematic literature review highlighted that gamification has been used as a highly relevant and effective strategy in education [44]. Morrover, Haruna et al. (2021) revealed that a serious gamified system played a positive role in enhancing instructional outcomes of students in a sexual health course through motivating and involving them in learning, improving their knowledge and inducing their attitude change by using the game mechanics [45]. As the proposed framework, computer simulations can improve and assess students’ skills. The experts indicated that the use of SGs or gamification can be appropriate solutions for enhancing the knowledge or theoretical aspects of medical education. In addition, the combination of computer simulation and digital game features can further encourage user engagement.

Concerning child abuse prevention, most studies have been conducted to prevent child sexual abuse using SGs [20, 25–29]. Additionally, Love et al. (2016) examined the feasibility of gamification-based parenting programs to help in preventing child abuse [15]. There is a need to provide programs for preventing child abuse outcomes [10]. In the healthcare field, SG can play a useful role in preventive goals by improving health-related information and behavioral change [46, 47]. Accordantly, Yant et al. (2019) showed that enjoyment was the most important feature of SGs for children [48]. The findings of a meta-analysis presented that short-term and long-term gamification-based intervention can be a promising method for initiating behavioral changes in learners [49]. In this study, experts stated that there is a need to provide educational programs for children, parents, and the community to prevent child abuse. The selection of appropriate IT-based solutions for this purpose depends on the age of the users. It appears that SGs in providing indirect education are a suitable way for children’s learning goals. Moreover, gamification by using game elements in non-game content can engage adults in the educational process.

There are various screening tools, i.e., questionnaires and checklists, for detecting child abuse cases [50–52]. Developing a safe non-intrusive tool can be a useful solution for parents and caregivers to detect potential CSA cases [30]. Accordantly, Amita (2016) designed a prototype storytelling game as a novel method to support children’s unfettered creative expression of their daily events and help to detect potential CSA [30]. In our proposed framework, the experts recommended that there is a need to develop a serious game-based screening tool for indirect screening of child abuse cases. Therefore, digital game-based tools have the potential to be used in child abuse fields for the screening objectives.

The results of this study demonstrate that no studies have used digital games or VR to help diagnose child maltreatment cases, but only a study has used the immersive VR to examine whether the level of professional experience and cognitive load of GPs affect the correct diagnosis of child safeguarding issues [21]. It seems that the use of mathematical models as a non-interactive type of computer simulation is a more suitable solution for diagnosing cases of child maltreatment. For example, Thompson and Bertocci (2013) [53], and Bertocci et al. (2001) [54] developed computer simulation models to study the biomechanics of pediatric bed fall, and stair falls, respectively. In this study, experts recommended that SGs can be used for questionnaire-based diagnosis of child abuse. Various studies should be conducted in support of this suggestion.

By providing nonverbal communication services, SGs could facilitate the treatment of child sexual abuse victims [31, 32]. Moreover, a video game has been developed to aid adopted children with trauma [33]. Within the proposed framework, digital games and VR can be used to treat various consequences of child abuse, such as psychological consequences (e.g., aggression, suicide, guilty feeling, depression, and PTSD), and problematic behavior of child abusers in cases of child abuse. Dewhirst et al. (2022) and Lau et al. (2017) revealed that SGs could be used as an effective tool for reducing mental health symptoms [55, 56]. Various papers indicated that SGs are efficient therapeutic solutions for rehabilitation objectives [57–59]. In the child abuse field, much more evidence is required to investigate the beneficial role of SGs in therapeutic purposes by conducting more original papers. It should be noted that any type of intervention aimed at preventing, screening, diagnosing, and treating cases of child abuse requires consideration of preventive solutions to retraumatization.

The findings of this study also showed that immersive VR is a more highlighted technology in forensic medicine by providing behavioral monitoring and risk assessment of sexual offenders [34, 35, 37, 38, 60]. Additionally, Davy-Jow et al. (2013) demonstrated that a non-immersive VR (graphical and interactive computer simulation) named virtual forensic anthropology was developed for educational purposes [36]. Our results are in agreement with those of Fromberger et al. (2018) study, showing the potential capabilities of VR in diagnosis, risk assessment, and therapy of child sexual offenders [40]. Furthermore, the experts recommended that the education of forensic medicine programs in cases of child abuse, including forensic pathology, clinical forensic medicine, forensic psychiatry, and forensic anthropology can be facilitated by using VR and computer simulations. Therefore, future research is needed to demonstrate the applications of VR in forensic medicine for child maltreatment cases.

## Implication of the study

The main theoretical implication of present study is that it gives a comprehensive review of the SGs and VR applications for child abuse issues, which serves as the conception for some future applications. As for the practical implications, the development of a conceptual framework can be considered by the government, decision-makers, psychologists, psychiatrists, digital game designers, health information technology experts, researchers, and other stackholders to decide about the use of SGs and VR solutions for the management of the child abuse issues in their plans. Another practical implication is the identification of suitable technologies (i.e., SGs, VR, computer simulations, and gamification) in terms of their applicability to the management of child abuse, thus setting the course for their further development and applications.

## Conclusion, limitation, and future work

Digital games and VR technologies have various applications in child abuse management, which have focused on six main categories: (1) medical education, (2) prevention, (3) screening, (4) diagnosis, (5) treatment, and (6) forensic medicine objectives. Serious game-based solutions have more used for the prevention and treatment of child abuse cases. Interestingly, non-immersive VR (i.e., interactive and graphical computer simulations), and immersive VR were more considered in medical education and forensic medicine, respectively. It appears that digital games and VR can be used as appropriate solutions to respond to child abuse requirements because of their capabilities. Consequently, planning for the realization of the mentioned technologies’ potential should be more considered by policymakers, stakeholders, and researchers.

The strengths of this study are its mixture method (i.e., a scoping review and expert knowledge), comprehensive scope, and search of multiple information sources. The major limitation of this paper is that it only includes articles in English. Therefore, it is recommended that in future studies, a multilingual research team conduct this work to address the mentioned limitation.

## Supporting information

S1 Table Preferred Reporting Items for Systematic reviews and Meta-Analyses extension for Scoping Reviews (PRISMA-ScR) checklist. (DOCX)

S2 Table The search strategy in PubMed. (DOCX)

S3 Table Digital games and VR based solutions employed in the child abuse concern. (DOCX)

S4 Table Technical aspects of the included papers. (DOCX)
