# Supplementary material for: Digital games and virtual reality applications in child abuse: A scoping review and conceptual framework
Source: PLoS One. 2022 Nov 9;17(11):e0276985. doi: 10.1371/journal.pone.0276985 (PMC9645636; doi:10.1371/journal.pone.0276985)
Supplement: S2 Table — (DOCX) [file pone.0276985.s002.docx]

**S2 Table.** The search strategy in PubMed

| ID | Details | Results |
| --- | --- | --- |
| 1 | "Child"[MeSH Terms] OR "Adolescent"[MeSH Terms] OR "Infant"[MeSH Terms] | [3,768,647](https://pubmed.ncbi.nlm.nih.gov/?term=%22Child%22%5BMeSH+Terms%5D+OR+%22Adolescent%22%5BMeSH+Terms%5D+OR+%22Infant%22%5BMeSH+Terms%5D&ac=no&sort=relevance) |
| 2 | "child*"[Title/Abstract] OR "adolescent"[Title/Abstract] OR "early life"[Title/Abstract] OR "infant"[Title/Abstract] OR "baby"[Title/Abstract] OR "teen*"[Title/Abstract] OR "youth*"[Title/Abstract] OR "school aged"[Title/Abstract] OR "young people"[Title/Abstract] OR "pre-school"[Title/Abstract] OR "preschool"[Title/Abstract] | [1,904,141](https://pubmed.ncbi.nlm.nih.gov/?term=%22child%2A%22%5BTitle%2FAbstract%5D+OR+%22adolescent%22%5BTitle%2FAbstract%5D+OR+%22early+life%22%5BTitle%2FAbstract%5D+OR+%22infant%22%5BTitle%2FAbstract%5D+OR+%22baby%22%5BTitle%2FAbstract%5D+OR+%22teen%2A%22%5BTitle%2FAbstract%5D+OR+%22youth%2A%22%5BTitle%2FAbstract%5D+OR+%22school+aged%22%5BTitle%2FAbstract%5D+OR+%22young+people%22%5BTitle%2FAbstract%5D+OR+%22pre-school%22%5BTitle%2FAbstract%5D+OR+%22preschool%22%5BTitle%2FAbstract%5D&ac=no&sort=relevance) |
| 3 | #1 OR #2 | [4,245,414](https://pubmed.ncbi.nlm.nih.gov/?term=%28%22child%2A%22%5BTitle%2FAbstract%5D+OR+%22adolescent%22%5BTitle%2FAbstract%5D+OR+%22early+life%22%5BTitle%2FAbstract%5D+OR+%22infant%22%5BTitle%2FAbstract%5D+OR+%22baby%22%5BTitle%2FAbstract%5D+OR+%22teen%2A%22%5BTitle%2FAbstract%5D+OR+%22youth%2A%22%5BTitle%2FAbstract%5D+OR+%22school+aged%22%5BTitle%2FAbstract%5D+OR+%22young+people%22%5BTitle%2FAbstract%5D+OR+%22pre-school%22%5BTitle%2FAbstract%5D+OR+%22preschool%22%5BTitle%2FAbstract%5D%29+OR+%28%22Child%22%5BMeSH+Terms%5D+OR+%22Adolescent%22%5BMeSH+Terms%5D+OR+%22Infant%22%5BMeSH+Terms%5D%29&ac=no&sort=relevance) |
| 4 | "abuse*"[Title/Abstract] OR "maltreat*"[Title/Abstract] OR "neglect*"[Title/Abstract] OR "molest*"[Title/Abstract] OR "advers*"[Title/Abstract] OR "pedophil*"[Title/Abstract] OR "paedophile*"[Title/Abstract] OR "assult*"[Title/Abstract] OR "batter*"[Title/Abstract] | [917,514](https://pubmed.ncbi.nlm.nih.gov/?term=%22abuse%2A%22%5BTitle%2FAbstract%5D+OR+%22maltreat%2A%22%5BTitle%2FAbstract%5D+OR+%22neglect%2A%22%5BTitle%2FAbstract%5D+OR+%22molest%2A%22%5BTitle%2FAbstract%5D+OR+%22advers%2A%22%5BTitle%2FAbstract%5D+OR+%22pedophil%2A%22%5BTitle%2FAbstract%5D+OR+%22paedophile%2A%22%5BTitle%2FAbstract%5D+OR+%22assult%2A%22%5BTitle%2FAbstract%5D+OR+%22batter%2A%22%5BTitle%2FAbstract%5D&ac=no&sort=relevance) |
| 5 | 3 AND 4 | [215,321](https://pubmed.ncbi.nlm.nih.gov/?term=%28%22abuse%2A%22%5BTitle%2FAbstract%5D+OR+%22maltreat%2A%22%5BTitle%2FAbstract%5D+OR+%22neglect%2A%22%5BTitle%2FAbstract%5D+OR+%22molest%2A%22%5BTitle%2FAbstract%5D+OR+%22advers%2A%22%5BTitle%2FAbstract%5D+OR+%22pedophil%2A%22%5BTitle%2FAbstract%5D+OR+%22paedophile%2A%22%5BTitle%2FAbstract%5D+OR+%22assult%2A%22%5BTitle%2FAbstract%5D+OR+%22batter%2A%22%5BTitle%2FAbstract%5D%29+AND+%28%28%22child%2A%22%5BTitle%2FAbstract%5D+OR+%22adolescent%22%5BTitle%2FAbstract%5D+OR+%22early+life%22%5BTitle%2FAbstract%5D+OR+%22infant%22%5BTitle%2FAbstract%5D+OR+%22baby%22%5BTitle%2FAbstract%5D+OR+%22teen%2A%22%5BTitle%2FAbstract%5D+OR+%22youth%2A%22%5BTitle%2FAbstract%5D+OR+%22school+aged%22%5BTitle%2FAbstract%5D+OR+%22young+people%22%5BTitle%2FAbstract%5D+OR+%22pre-school%22%5BTitle%2FAbstract%5D+OR+%22preschool%22%5BTitle%2FAbstract%5D%29+OR+%28%22Child%22%5BMeSH+Terms%5D+OR+%22Adolescent%22%5BMeSH+Terms%5D+OR+%22Infant%22%5BMeSH+Terms%5D%29%29&ac=no&sort=relevance) |
| 6 | "Child Abuse"[MeSH Terms] OR "child abuse, sexual"[MeSH Terms] OR "Emotional Abuse"[MeSH Terms] OR "Physical Abuse"[MeSH Terms] OR "Sexual Trauma"[MeSH Terms] OR "child, foster"[MeSH Terms] | [33,146](https://pubmed.ncbi.nlm.nih.gov/?term=%22Child+Abuse%22%5BMeSH+Terms%5D+OR+%22child+abuse%2C+sexual%22%5BMeSH+Terms%5D+OR+%22Emotional+Abuse%22%5BMeSH+Terms%5D+OR+%22Physical+Abuse%22%5BMeSH+Terms%5D+OR+%22Sexual+Trauma%22%5BMeSH+Terms%5D+OR+%22child%2C+foster%22%5BMeSH+Terms%5D&ac=no&sort=relevance) |
| 7 | 5 OR 6 | [224,502](https://pubmed.ncbi.nlm.nih.gov/?term=%28%22Child+Abuse%22%5BMeSH+Terms%5D+OR+%22child+abuse%2C+sexual%22%5BMeSH+Terms%5D+OR+%22Emotional+Abuse%22%5BMeSH+Terms%5D+OR+%22Physical+Abuse%22%5BMeSH+Terms%5D+OR+%22Sexual+Trauma%22%5BMeSH+Terms%5D+OR+%22child%2C+foster%22%5BMeSH+Terms%5D%29+OR+%28%28%22abuse%2A%22%5BTitle%2FAbstract%5D+OR+%22maltreat%2A%22%5BTitle%2FAbstract%5D+OR+%22neglect%2A%22%5BTitle%2FAbstract%5D+OR+%22molest%2A%22%5BTitle%2FAbstract%5D+OR+%22advers%2A%22%5BTitle%2FAbstract%5D+OR+%22pedophil%2A%22%5BTitle%2FAbstract%5D+OR+%22paedophile%2A%22%5BTitle%2FAbstract%5D+OR+%22assult%2A%22%5BTitle%2FAbstract%5D+OR+%22batter%2A%22%5BTitle%2FAbstract%5D%29+AND+%28%28%22child%2A%22%5BTitle%2FAbstract%5D+OR+%22adolescent%22%5BTitle%2FAbstract%5D+OR+%22early+life%22%5BTitle%2FAbstract%5D+OR+%22infant%22%5BTitle%2FAbstract%5D+OR+%22baby%22%5BTitle%2FAbstract%5D+OR+%22teen%2A%22%5BTitle%2FAbstract%5D+OR+%22youth%2A%22%5BTitle%2FAbstract%5D+OR+%22school+aged%22%5BTitle%2FAbstract%5D+OR+%22young+people%22%5BTitle%2FAbstract%5D+OR+%22pre-school%22%5BTitle%2FAbstract%5D+OR+%22preschool%22%5BTitle%2FAbstract%5D%29+OR+%28%22Child%22%5BMeSH+Terms%5D+OR+%22Adolescent%22%5BMeSH+Terms%5D+OR+%22Infant%22%5BMeSH+Terms%5D%29%29%29&ac=no&sort=relevance) |
| 8 | "Virtual Reality"[MeSH Terms] OR "Virtual Reality Exposure Therapy"[MeSH Terms] OR "Augmented Reality"[MeSH Terms] OR "Video Games"[MeSH Terms] OR "computer simulation"[MeSH Terms] | [272,615](https://pubmed.ncbi.nlm.nih.gov/?term=%22Virtual+Reality%22%5BMeSH+Terms%5D+OR+%22Virtual+Reality+Exposure+Therapy%22%5BMeSH+Terms%5D+OR+%22Augmented+Reality%22%5BMeSH+Terms%5D+OR+%22Video+Games%22%5BMeSH+Terms%5D+OR+%22computer+simulation%22%5BMeSH+Terms%5D&ac=no&sort=relevance) |
| 9 | "virtual reality"[Title/Abstract] OR "video game*"[Title/Abstract] OR "game*"[Title/Abstract] OR "serious game*"[Title/Abstract] OR "gamification"[Title/Abstract] OR "exergame"[Title/Abstract] OR "augmented reality"[Title/Abstract] OR "mixed reality"[Title/Abstract] OR "computer* simulat*"[Title/Abstract] | [107,687](https://pubmed.ncbi.nlm.nih.gov/?term=%22virtual+reality%22%5BTitle%2FAbstract%5D+OR+%22video+game%2A%22%5BTitle%2FAbstract%5D+OR+%22game%2A%22%5BTitle%2FAbstract%5D+OR+%22serious+game%2A%22%5BTitle%2FAbstract%5D+OR+%22gamification%22%5BTitle%2FAbstract%5D+OR+%22exergame%22%5BTitle%2FAbstract%5D+OR+%22augmented+reality%22%5BTitle%2FAbstract%5D+OR+%22mixed+reality%22%5BTitle%2FAbstract%5D+OR+%22computer%2A+simulat%2A%22%5BTitle%2FAbstract%5D&ac=no&sort=relevance) |
| 10 | 8 OR 9 | [356,691](https://pubmed.ncbi.nlm.nih.gov/?term=%28%22virtual+reality%22%5BTitle%2FAbstract%5D+OR+%22video+game%2A%22%5BTitle%2FAbstract%5D+OR+%22game%2A%22%5BTitle%2FAbstract%5D+OR+%22serious+game%2A%22%5BTitle%2FAbstract%5D+OR+%22gamification%22%5BTitle%2FAbstract%5D+OR+%22exergame%22%5BTitle%2FAbstract%5D+OR+%22augmented+reality%22%5BTitle%2FAbstract%5D+OR+%22mixed+reality%22%5BTitle%2FAbstract%5D+OR+%22computer%2A+simulat%2A%22%5BTitle%2FAbstract%5D%29+OR+%28%22Virtual+Reality%22%5BMeSH+Terms%5D+OR+%22Virtual+Reality+Exposure+Therapy%22%5BMeSH+Terms%5D+OR+%22Augmented+Reality%22%5BMeSH+Terms%5D+OR+%22Video+Games%22%5BMeSH+Terms%5D+OR+%22computer+simulation%22%5BMeSH+Terms%5D%29&ac=no&sort=relevance) |
| 11 | 10 AND 7 | [1,074](https://pubmed.ncbi.nlm.nih.gov/?term=%28%28%22virtual+reality%22%5BTitle%2FAbstract%5D+OR+%22video+game%2A%22%5BTitle%2FAbstract%5D+OR+%22game%2A%22%5BTitle%2FAbstract%5D+OR+%22serious+game%2A%22%5BTitle%2FAbstract%5D+OR+%22gamification%22%5BTitle%2FAbstract%5D+OR+%22exergame%22%5BTitle%2FAbstract%5D+OR+%22augmented+reality%22%5BTitle%2FAbstract%5D+OR+%22mixed+reality%22%5BTitle%2FAbstract%5D+OR+%22computer%2A+simulat%2A%22%5BTitle%2FAbstract%5D%29+OR+%28%22Virtual+Reality%22%5BMeSH+Terms%5D+OR+%22Virtual+Reality+Exposure+Therapy%22%5BMeSH+Terms%5D+OR+%22Augmented+Reality%22%5BMeSH+Terms%5D+OR+%22Video+Games%22%5BMeSH+Terms%5D+OR+%22computer+simulation%22%5BMeSH+Terms%5D%29%29+AND+%28%28%22Child+Abuse%22%5BMeSH+Terms%5D+OR+%22child+abuse%2C+sexual%22%5BMeSH+Terms%5D+OR+%22Emotional+Abuse%22%5BMeSH+Terms%5D+OR+%22Physical+Abuse%22%5BMeSH+Terms%5D+OR+%22Sexual+Trauma%22%5BMeSH+Terms%5D+OR+%22child%2C+foster%22%5BMeSH+Terms%5D%29+OR+%28%28%22abuse%2A%22%5BTitle%2FAbstract%5D+OR+%22maltreat%2A%22%5BTitle%2FAbstract%5D+OR+%22neglect%2A%22%5BTitle%2FAbstract%5D+OR+%22molest%2A%22%5BTitle%2FAbstract%5D+OR+%22advers%2A%22%5BTitle%2FAbstract%5D+OR+%22pedophil%2A%22%5BTitle%2FAbstract%5D+OR+%22paedophile%2A%22%5BTitle%2FAbstract%5D+OR+%22assult%2A%22%5BTitle%2FAbstract%5D+OR+%22batter%2A%22%5BTitle%2FAbstract%5D%29+AND+%28%28%22child%2A%22%5BTitle%2FAbstract%5D+OR+%22adolescent%22%5BTitle%2FAbstract%5D+OR+%22early+life%22%5BTitle%2FAbstract%5D+OR+%22infant%22%5BTitle%2FAbstract%5D+OR+%22baby%22%5BTitle%2FAbstract%5D+OR+%22teen%2A%22%5BTitle%2FAbstract%5D+OR+%22youth%2A%22%5BTitle%2FAbstract%5D+OR+%22school+aged%22%5BTitle%2FAbstract%5D+OR+%22young+people%22%5BTitle%2FAbstract%5D+OR+%22pre-school%22%5BTitle%2FAbstract%5D+OR+%22preschool%22%5BTitle%2FAbstract%5D%29+OR+%28%22Child%22%5BMeSH+Terms%5D+OR+%22Adolescent%22%5BMeSH+Terms%5D+OR+%22Infant%22%5BMeSH+Terms%5D%29%29%29%29&ac=no&sort=relevance) |
