# Supplementary material for: Digital games and virtual reality applications in child abuse: A scoping review and conceptual framework
Source: PLoS One. 2022 Nov 9;17(11):e0276985. doi: 10.1371/journal.pone.0276985 (PMC9645636; doi:10.1371/journal.pone.0276985)
Supplement: S3 Table — (DOCX) [file pone.0276985.s003.docx]

**S3 Table.**  Digital games and VR based solutions employed in the child abuse concern

| **Classification** | **Authors (year)** | **Country** | **Child abuse type** | **Product type** | **Aim** | **Total number of subjects** | **Study design** | **Mean age of samples (M), standard deviation (SD)** | **Assessment variables: Assessment tools** | **Intervention duration** | **main results** |
| --- | --- | --- | --- | --- | --- | --- | --- | --- | --- | --- | --- |
| **Medical education** | Zhao et al. (2019) [22] | USA | Physical abuse | Computer simulation | Designing a game to assess child physical abuse symptoms | 100 | Single group: Undergraduate students | M= 19.8 years, SD= 2.70 | - The comfort of users: Asked questions - Knowledge assessment: Feature selection | One time (details: NA) | - The students reported that the Computer Simulated Interactive Child Abuse Screening Tool (CSI-CAST) was easy to use (M= 8.6, SD= 1.64). - The CSI-CAST has used assistive AI technologies to assess the knowledge of students and discover features that are important in identifying child physical abuse. |
|  | McEvoy et al.(2011) [23] | Ireland | Physical abuse | Computer simulation | Developing an interactive video-based Virtual Patient for formal training to recognize suspected cases of child maltreatment | 25 | Single group: Pediatrics Basic Specialist Trainees | NA | Perception of users: A researcher-made questionnaire | One time (details: NA) | - The students indicated that the virtual patient was an enjoyable, interactive, and appropriate educational approach. - Ninety-two percent of samples indicated greater self-confidence in their ability to diagnose cases of suspected child physical abuse. - Ninety-six percent of samples indicated greater self-confidence in their ability to report cases of suspected child physical abuse. |
|  | Dorsey et al. (1996) [24] | USA | Sexual abuse | Computer simulation | Investigating the medical student responses to an interactive patient simulation system used to supplement education in child maltreatment | 40 | Single group: Freshmen medical students | NA | - Student attitudes and opinions: A questionnaire - Student performance: Correct diagnosis - Indicators of academic achievement: Medical College Admission Test (MCAT) scores and college grade point average (GPA)) | One time (details: NA) | - All students reported that computer simulation was an interesting and educational tool. - The correct diagnosis was made by 29 students - There were no significant differences in GPA or MCAT scores between gender or experience groups |
|  | Kost, S. and  Schwartz, W (1989) [19] | USA | Physical and sexual abuse, patient neglect | Computer simulation | Evaluating the skills of medical students in correcting diagnosis of child abuse cases using computer simulation. | 81 | Two groups of medical students:  *Experimental group (EXP): N= 38  *Control group (CG): N= 43 | NA | - Examination: Score | The three-hour seminar | - In the classic case of child abuse (sore arm): Two groups had similar results (CG: score= 62, cost= 398$, and EXP: score= 65. Cost: 332$) - In the more difficult diagnostic problem (shaken baby): The results of groups were significant. (EXG: score=76, cost= 599$, and CG: score= 68, cost= 806$) |
| **Prevention** | Malamsha et al (2021) [20] | Tanzania | Sexual abuse | Mobile-based game | Designing and validating a mobile-based serious game for educating prevention of CSA in Tanzania | ***Phase 1**: N= 135  ***Phase 2**: N= 37 | **Game development consists of 3 phases:**  Preproduction, production, and post-production  ****Phase 1:** **Determine the content of the game**:  *Parents: N= 111  *Child experts: N= 24  ****Phase 2:**  **Validation of game:**  *****Parents: N= 32  *Children: N= 5 | ***Phase 1:** parents with  at least 1 child aged 1 to 18 years    ***Phase 2**: parents with at least 1 child aged 3 to 5 years | - Parents and caretakers’ requirements: Online survey, a paper questionnaire - Child expert requirements: Focus group meeting - Game validation: A survey: questionnaire, interviews, and observations | From March 2020 to April 2020  +  Between February 2021 and March 2021 | - Out of 32 interviewed, 81% (N= 26) scored as a “satisfied parent”. - A score of 4 out of 5 (“somewhat satisfied”) was given by 19% of parents (N= 6). - The confidence level of parents in talking about CSAP increased (before using the game: 3.56 (neutral), after using the game: 4.9 (confident)). - All five children were interested and enjoyed during the game. - The ability scores, calculated based on a range of topics included in CSAP education talks with children, also increased from 5.67 (out of 10) to 8.8 (out of 10) after the game was played. |
|  | Jones et al. (2020) [25] | Australia | Sexual abuse | Computer game | Designing and evaluating a game for the prevention of CSA in Australia | 126 | Three groups of elementary school students:  *CG: N= 33  *Game only Group: N=45  *Game and Lesson Group: N=48 | M= 9.7 years, SD= NA | The knowledge of abuse prevention: Children’s Knowledge of Abuse  Questionnaire-Revised (CKAQ-R-III) test and CKAQ Short form | To be played over a 5–10 week period (game time: 6h) | - The Game Group and Game and Lesson Group had increased their scores on CKAQ (non-significant increases of 2.42 ± 0.58 and 2.67 ± 0.47 respectively for 31 items, and significant increases of 1.20 ± 0.28 and 1.54 ± 0.29 for the short form 17 item version). - Students' knowledge about preventing sexual abuse increased significantly for students who completed the game compared to those who did not. - Students in the control group who did not use the game did not significantly increase their CKAQ scores. |
|  | Shan, Yushinan (2019) [26] | USA | Sexual abuse | Game | Designing an educational mobile-based game to help and prevent child sexual abuse. | 20 | *Game development  * **Imagine Rochester Institute of Technology (RIT)**: Parents and children: N= 20  (details are not applicable) | NA | Imagine RIT: A questionnaire | NA | The most of users gave positive ideas about the game (e.g., Easy navigation, satisfaction with design and style, and appropriateness of content) |
|  | Love et al. 2016 [15] | USA | Parenting program | Gamification | Examining the feasibility and the efficacy of an online parenting program called Triple P Online (TPOC) | 155 | A single intervention group: Disadvantaged, high-risk parents | M= 33 years, SD= 7.5 | - Children’s externalizing behaviors: The Eyberg Child Behavior Inventory (ECBI) - parents’ dysfunctional parenting style: The Parenting Scale - Parents’ self-efficacy in managing child's emotional and behavioral problems: CAPES Self-efficacy scale - Parents’ negative attributional style: The Parent’s Attributions for Child’s Behavior Measure (PACBM) - Parents’ adjustment difficulties: The Depression Anxiety Stress Scales (DASS-21) - Patterns of Program Use: TPOC’s content management system - Satisfaction with the Program: Five-point scale questionnaire - Parents’ feedback for the system: Focus group - Agency-level barriers: Agency Exit Interviews | Twelve weeks + 6 months follow up | - Both the ECBI intensity and the CAPES behavior scale showed a significant reduction in problem behavior. - The CAPES emotion scale showed a significant reduction in emotional problems - No effects were found on the ECBI Problem scale, CAPES parental confidence, PS Verbosity, PACBM attributional measures, DASS, depression, and anxiety scales. - Consumer satisfaction with the program, including social media and gaming elements, was strong. - Running out of time was reported as the most common barrier. - Parents’ overall experience of TPOC was very positive - Gaming aspects also enhanced motivation. |
|  | Gilliam et al. (2016) [27] | USA | Sexual violence | Digital Game | Designing an interactive game to improve learning and communication about sexual violence | 24 | Single group: Young people | *M and SD: NA  *Age ranges: 14 to 18 years (Median= 17 years) | Participants' opinions: A post-game focus group and follow-up interviews | Two times (each time: 45 minutes) | Participants in the study agreed with the game and reported increased interest in sexuality education, public awareness, and communication about sexual violence and health issues, particularly at school. |
|  | Müller et al. (2014) [28] | Germany | sexual abuse | Gamification | Developing a web-based training system with gamification features for CSA prevention | 286 | Two groups of children:  *EXP: N= 137  *CG: N= 149 | M= 9 years, SD= 0.76 | - Knowledge (the Children's Knowledge of Abuse Questionnaire) - Behavioral intentions (Four situations that would allow for risky or secure behaviors were presented to the children) - Anxiety (specific Anxiety Questionnaire) - Emotion regulation (child sexual abuse the Emotion Awareness Questionnaire) - Children’s acceptance of “Cool and Safe” (score) | *Two times (during 4 weeks)  * The completion of the whole program takes about two hours | - Knowledge and training secure behavioral strategies improved in the EXP group - No increased levels of anxiety were detected - A decrease in emotional concealment was found in children who participated in the training compared to a control group. |
|  | Jones (2008) [29] | Australia | Sexual abuse | Game | Designing an online game for educating anti-abduction and sexual abuse prevention strategies for children aged 6 to 8 | NA | Game design and implementation stage | NA | NA | NA | The key safety awareness messages have presented in this game include asking parents first, sticking with your buddy, watching where you are going, and trusting friends |
| **Screening** | Amita R Pharshy (2016) [30] | Canada | Sexual abuse | Game | Developing a prototype storytelling game to support children's unfettered creative expression of their daily events and help to detect potential CSA | 23 | Design a prototype of a storytelling app: Parents, caregivers, and other adults were invited to determine cross-cultural notions around CSA and design elements | 18 years and above | Cultural aspects of adults’ approach to CSA and design elements (an electronic survey questionnaire) | Four months | - All participants were concerned about CSA. - Twenty-two participants reported that they would like to educate their children about CSA. - Nineteen participants reported that if there were a resource available to educate children about sexual abuse, they would expose their children to use it. - Seventeen participants suggested storytelling as a good solution for detecting potential CSA. - The prototype of a children’s storytelling app was developed for detecting potential child sexual abuse. |
| **Diagnosis** | Pan et al. (2018) [21] | United Kingdom | Child safeguarding concerns | VR | Designing the immersive VR program to examine whether the level of professional experience and cognitive load of GPs affect the correct diagnosis of child safeguarding issues | 63 | Single group:  (GPs): N= 37 + Trainee GPs: N= 27 | *M and SD: NA  *Age ranges: 25-59 | Responses of GPs/ the doctor notes left on the laptop immediately after the consultation: The average of the 10 ratings of evaluators | One time | - No relationship was found between the experience of GPs and the quality of their notes. - GPs with less stress and less neurotic tend to be better at reporting potential child abuse cases in their notes. |
| **Treatment** | Endendijk et al. (2021) [31] | Netherlands | Sexual abuse | Serious game | Determining which therapy component of the developed serious game was used by the therapist | 10 | Single group: Therapists who were using the game during the study | M= 42.7, SD= NA | Therapeutic components used by therapists: Online questionnaires and semi-structured interviews | Therapists completed the questionnaire each time they used the serious game | - The game was used by all 10 therapists and for about 60% of clients to facilitate a narrative of traumatic CSA experiences. - The game was used by about one-third of clients to provide psycho-education. - Six therapists had used or would use the developed serious game to learn sex-related vocabulary. |
|  | Johanna et al (2021) [32] | Netherlands | Sexual abuse | Serious game | * Determining which working components of the serious game could be recognized in therapy with victims of sexual abuse.  * Determining how the acceptability of the serious game was evaluated by therapists | 10 | Single group: Therapists who were using the game during the study | M= 42.7 years, SD= NA | Working Elements and Therapist Acceptability of the game: Web-based questionnaire | Therapists completed the questionnaire each time they used Vil Du?! | - The game enabled the child to control the situation (90% of answers) - Most therapists (in 35% of the questionnaires and 80% of the interviews) reported that the serious game has reduced barriers to the disclosure of CSA experiences for their clients. - The playful environment of the serious game as a working element has been mentioned by 60% of therapists in the interviews - Time efficiency was reported by 80% of therapists in the interviews. - Fifty percent of therapists reported by using the serious game could obtain more information regarding the client’s CSA experience. - The questionnaire data demonstrated that all aspects of client satisfaction resulted in average scores higher than the neutral mid-point of the scale. |
|  | Sallot (2021) [33] | USA | Trauma, abuse and neglect | Video game | Designing the video game to aid adopted children with trauma. | 3 | Game design and concept testing via participants who worked with adopted children:  *Play therapists: N= 2  *Social worker: N= 1 | NA | Concept testing (viability of the game within the therapeutic space): Interview | One time | Feedback on the game and general concept was positive. |
| **Forensics Research** | Fromberger et al. (2018) [34] | Germany | Sexual offenders against children (SOC) | VR | Testing the potential of VR for SOCs behavioral monitoring in high-risk situations | 13 | Two groups:  *Sexual offender: N= 6  *Non-offender controls (NOC): N= 7 | *SOCs: M= 47.67 years, SD= 13.47  *NOCs: M= 26.00 years, SD= 4.36 | - To measure typical symptoms of simulator sickness: The Simulator Sickness Questionnaire - To measure the sense of presence in VR environments: The Igroup Presence Questionnaire - To measure the sense of co-presence: The Igroup Presence Questionnaire - To measure the simulation realism: The German VR Simulation Realism Scale | One time | - The subjective feeling of reported presence was elevated in every experimental condition. - The subjective co-presence was at a medium level in all conditions for both groups of participants. - The reported severity of simulator-based disease symptoms was low following each experimental condition in both groups of participants. - The Scene Realism factor showed differences between the two groups under all experimental conditions with low to medium effect sizes. |
|  | Renaud et al.(2015) [35] | Canada | Sexual arousal responses | VR | Comparing the respective sexual arousal responses and the perceptualmotor patterns of child molesters and sexually non-deviant (ND) male | 42 | Two groups:  *Male child molesters: N= 13  *ND male subjects: N= 29 | * Child molesters group: M= 42 years, SD= 10.8  * ND male group: M= 41.9 years, SD= 10.4 | - Variations of blood volume in the penis: Circumferential penile plethysmography (PPG) - Gaze behavior dynamics: Virtual measurement points (VMPs) placed over virtual objects, derived from average gaze radial angular deviation (GRAD) and GRAD coefficient of variation (GRADCV) | *Five 90-second periods  * The entire procedure lasted on average 1 hour and 30 minutes, including reception, screening questionnaires, and debriefing. | The results showed distinct patterns of sexual arousal depending on sexual preferences and pointed toward the existence of specific gaze behavior dynamics guided by sexual intentions. |
|  | Renaud et al.(2014) [36] | Canada | Deviant sexual preferences | VR | Determining how the VR modality compared to the standard auditory modality on their capacity to generate sexual arousal. | 64 | Apply both VR and the standard auditory modality interventions to participants:  * sex offenders of children (male): N= 22  * non-deviant adult males (male): N= 42 | N=64:  *Clinical group: M= 43.5 years, SD= 13.7)  *The control group: M= 40.7 years, SD= 11.5 | The measurement of blood flow variations in the penis during sexual arousal: PPG | The entire procedure was approximately 2 h | The VR modality provided significantly better group classification accuracy and discriminant validity compared to audio stimuli |
|  | Davy-Jow et al. (2013) [37] | USA | Neglect | Computer simulation | Developing virtual forensic anthropology in a child death case | One girl | Generating full-body 3D virtual reconstructions (Case report) | 7 years old | NA | NA | Accurate representations of maltreatment and neglect in the case of a girl's death have been documented in 3D. |
|  | Renaud et al. (2010) [38] | Canada | Forensic assessment of sex offenders | VR | Designing immersive virtual characters for forensic assessment of sex offenders | 25 | Two groups:  * Sexual aggressors against children (SAAC): N= 10  * Non-deviant (ND) male subjects: N= 15 | *SAAC group: M= 39.9 years, SD= 13.6  *ND group: M= 45.5 years, SD 11.0 | Variations in sexual organs’ blood volume: PPG | Five 120 second periods | - The ND subjects showed more sexual arousal when facing virtual characters simulating adult. - The SAACs responded more strongly to virtual characters mimicking sexual features belonging to children aged 10-12 years. |
|  | Renaud et al. (2009)  [39] | Canada | Diagnostic index of sexual deviancy | VR | Assessing gaze behavior nonlinear dynamics in the virtual immersion world | 16 | Two groups:  *Male pedophile patients: N= 8  *Male non-deviant control subjects: N= 8 | *Pedophile patients: M= 38.8 years, SD= 9.20  *Control subjects: M= 42.7 years, SD= 10.7 | - Variations in sexual organs’ blood volume: PPG - Eye-movement: Infrared diode whose signal is picked up by a small video camera - Gaze measurement: Six degrees of freedom (DOF) resulting from head movements and the two DOF (x and y coordinates) resulting from eye movements tracked by the eye-tracking system | For three 120 second periods | - Pedophile patients showed gaze behavior dynamics of lesser fractal complexity. - Pedophile patients showed higher relative erectile responses than normal subjects. |
