# Supplementary material for: Digital games and virtual reality applications in child abuse: A scoping review and conceptual framework
Source: PLoS One. 2022 Nov 9;17(11):e0276985. doi: 10.1371/journal.pone.0276985 (PMC9645636; doi:10.1371/journal.pone.0276985)
Supplement: S4 Table — (DOCX) [file pone.0276985.s004.docx]

**S4 Table.** Technical aspects of the included papers

| Classification | **Authors (year)** | Product type (game-based or non-game) | Product name | The product was designed in the paper or not | Availability of products for us (link) | Software requirements for developing | Hardware requirement | Scenario/content of the game |
| --- | --- | --- | --- | --- | --- | --- | --- | --- |
| **Medical education** | Zhao et al. (2019) [22] | Non-game (computer simulation) | The Computer Simulated Interactive Child Abuse Screening Tool (CSI-CAST) | Yes | Not available | - Unreal game engine - Programming language: C++, Blueprints - Machine learning (feature selection) | Computer | - The scenario presents a simulated scene of an examination room with an adult guardian (mom) and a child patient (Ben) that speak realistically in such a scenario - There are 51 binary choices in the scenario: - 23 questions for the child - 27 questions the user can choose to ask the mom - one interaction choice for the user (physical examination with the parent in the room or not) |
|  | McEvoy et al.(2011) [23] | Non-game (computer simulation) | Virtual patient | Yes | Not available | NA | Computer  (Details: NA) | A story about the physical abuse of a 7-month-old. |
|  | Dorsey et al. (1996) [24] | Non-game (computer simulation) | Diagnostic Reasoning (DxR) patient simulation computer program (Keyboard Publishing, Blue Bell, PA). | No | Not available | NA | Computer | A story about the sexual abuse of a 6-year-old girl. |
|  | Kost, S. and  Schwartz, W (1989) [19] | Non-game (computer simulation) | The CAMPS system (DACIS Software) | No | Not available | NA | Computer | Students can follow any path in the selection of more than 500 items of history, physical examination, laboratory, and treatments. |
| **Prevention**  **Prevention** | Malamsha et al (2021) [20] | Serious-game | HappyToto | Yes | Not available | - A flutter platform - Dart, a Google-developed object programming language - Adobe Illustrator software | Smartphone | The game consists of 3 levels: private parts, presents or gifts, and a safe environment |
|  | Jones et al. (2020) [25] | Serious-game | Name (Orbit) | Yes | Available:  orbit.org.au | NA | Computer | The game addresses ten key concepts related to CSA prevention. These include establishing a support network composed of five trustworthy adults from various segments of a child's life, understanding which parts of the body are private, knowing that it is wrong for others to touch or look at their private parts (body rules), tactics used by perpetrators, say and continue to say, and barriers to say. |
|  | Shan, Yushinan (2019) [26] | Game | Protect me | Yes | Not available | NA | Smartphone | - Teaching children what the private part is. - Teaching children what dangerous situations are and how to react to these situations., |
|  | Love et al. 2016 [15] | Gamification | Triple P Online (TPOL) | NO | NA | NA | Smartphone | TPOL has eight modules for an interactive self-directed positive parenting program. |
|  | Gilliam et al. (2016) [27] | Game | Lucidity | Yes | Not available | NA | Computer | The player discovers parts of the story about a woman who grapples with a sexual assault from her past. |
|  | Müller et al. (2014) [28] | Gamification | Cool and Safe | Yes | Available:  www. coolandsafe.eu | NA | Computer | Teaching safe behaviors, appropriate and inappropriate touches, and good/bad secrets |
|  | Jones (2008) [29] | Game | NA | Yes | Not available | NA | Computer | The main safety awareness messages are presented in eight distinct levels, including 1) ask parents first, 2) stick with your buddy, 3) watch where you are going, 4) trusted friends, 5) finding help if you’re lost, 5) Knowing, using your codeword, 6) Being safe when out and about, and 8) It’s okay to shout and tell |
| **Screening** | Amita R Pharshy (2016) [30] | Game | Storytelling game | Yes | Available:  http://rjyyfk.axshare.com/home.html. | Axure | Computer | In the game, children can create new stories of their own experiences through images, drawings, texts, and avatars created by themselves. |
| **Diagnosis** | Pan et al. (2018) [21] | Non-game (CAVE-like virtual reality system) | NA | Yes | Not available | - Unity3D - MiddleVR middleware | - Projectors - Active stereo glasses (Volfoni glasses) - 6-camera ART TrackPack system | A virtual consultation was presented in a CAVE-like system. |
| **Treatment** | Endendijk et al. (2021) [31] | Game | Vil Du?! | No | Available (commercial) | NA | Tablet | A nonverbal communication serious game that is similar to a dress-up game where children can demonstrate to the therapists what happened to them |
|  | Johanna et al (2021) [32] | Game | Vil Du?! | No | Available (commercial) | NA | Tablet | A nonverbal communication serious game that is similar to a dress-up game where children can demonstrate to the therapists what happened to them |
|  | Sallot (2021) [33] | Game | Mythos | Yes | Not available | - Adobe XD - Photoshop | Smartphone | In the game, adopted and traumatized children play the role of a stuffed bear who has lost his home and finds himself on a journey of self-discovery. |
| **Forensic medicine** | Fromberger et al. (2018) [34] | Non-game | NA | Yes | Not available | - PPT Wand (WorldViz LLC) - the 2D graphical software tool GIMP - the software tool Audacity - 3ds Max | - Head-mounted-display (HMD) - Motion capturing system (MoCap | A virtual supermarket was designed for the risk situations. |
|  | Renaud et al.(2015) [35] | Non-game | NA | Yes | Not available | NA | - HMD - A CAVE-type immersive system | The virtual characters have been used as sexual stimuli |
|  | Renaud et al.(2014) [36] | Non-game | NA | Yes | Not available | NA | - HMD - Infrared ocular tracking system | - The computer-generated stimuli are 3D virtual characters representing realistic naked human beings - A neutral scenario and two scenarios were presented, each representing five distinct categories of human interactions. |
|  | Davy-Jow et al. (2013) [37] | Non-game | NA | Yes | Not available | - A suite of 3D modeling engineering software packages (InnovMetric Inc.1 PolyWorks1, Topogun1) - Autodesk 3D Studio Max1 - Leica Cloudworx1, Bentley MicroStation1, - Autodesk AutoCAD1 - and Autodesk 3D Studio Max1 - 3Ds Max1 3D modeling package - MakeHuman1 software | - 3D laser scans - Hasselblad1 H3D 39-megapixel digital camera - A Leica1 TCR1105 total station theodolite and a Leica1 HDS6000 phase-based laser scanner | An accurate and interactive digital model of the deceased child was designed. |
|  | Renaud et al. (2010) [38] | Non-game | NA | Yes | Not available | A modeling software whose rationale is based on morphism | - A CAVE-type immersive system - A motion tracker - HMD | The 3D virtual characters were presented to depict a realistic naked human being |
|  | Renaud et al. (2009)  [39] | Non-game | NA | Yes | Not available | NA | - A CAVE-type immersive system - Active Nuvision 60GX stereoscopic glasses coupled with an oculomotor tracking system - Motion tracker | The 3D virtual characters were presented to depict a realistic naked human being |
